# The Night-Time Sleep and Autonomic Activity of Male and Female Professional Road Cyclists Competing in the Tour de France and Tour de France Femmes

**DOI:** 10.1186/s40798-024-00716-6

**Published:** 2024-04-16

**Authors:** Charli Sargent, Summer Jasinski, Emily R. Capodilupo, Jeremy Powers, Dean J. Miller, Gregory D. Roach

**Affiliations:** 1 CQUniversity, Appleton Institute for Behavioural Science, Adelaide, SA Australia; 2 Whoop Inc, Data Science and Research, Boston, MA USA

**Keywords:** Sleep duration, Recovery, Competition, Athletes, Exercise, Wearables, Heart rate variability

## Abstract

**Background:**

Sleep is a critical component of recovery, but it can be disrupted following prolonged endurance exercise. The objective of this study was to examine the capacity of male and female professional cyclists to recover between daily race stages while competing in the 2022 Tour de France and the 2022 Tour de France Femmes, respectively. The 17 participating cyclists (8 males from a single team and 9 females from two teams) wore a fitness tracker (WHOOP 4.0) to capture recovery metrics related to night-time sleep and autonomic activity for the entirety of the events and for 7 days of baseline before the events. The primary analyses tested for a main effect of ‘stage classification’—i.e., rest, flat, hilly, mountain or time trial for males and flat, hilly or mountain for females—on the various recovery metrics.

**Results:**

During baseline, total sleep time was 7.2 ± 0.3 h for male cyclists (mean ± 95% confidence interval) and 7.7 ± 0.3 h for female cyclists, sleep efficiency was 87.0 ± 4.4% for males and 88.8 ± 2.6% for females, resting HR was 41.8 ± 4.5 beats·min^−1^ for males and 45.8 ± 4.9 beats·min^−1^ for females, and heart rate variability during sleep was 108.5 ± 17.0 ms for males and 119.8 ± 26.4 ms for females. During their respective events, total sleep time was 7.2 ± 0.1 h for males and 7.5 ± 0.3 h for females, sleep efficiency was 86.4 ± 1.2% for males and 89.6 ± 1.2% for females, resting HR was 44.5 ± 1.2 beats·min^−1^ for males and 50.2 ± 2.0 beats·min^−1^ for females, and heart rate variability during sleep was 99.1 ± 4.2 ms for males and 114.3 ± 11.2 ms for females. For male cyclists, there was a main effect of ‘stage classification’ on recovery, such that heart rate variability during sleep was lowest after mountain stages. For female cyclists, there was a main effect of ‘stage classification’ on recovery, such that the percentage of light sleep (i.e., lower-quality sleep) was highest after mountain stages.

**Conclusions:**

Some aspects of recovery were compromised after the most demanding days of racing, i.e., mountain stages. Overall however, the cyclists obtained a reasonable amount of good-quality sleep while competing in these physiologically demanding endurance events. This study demonstrates that it is now feasible to assess recovery in professional athletes during multiple-day endurance events using validated fitness trackers.

**Supplementary Information:**

The online version contains supplementary material available at 10.1186/s40798-024-00716-6.

## Key Points

- Some aspects of recovery were compromised in male and female cyclists after mountain stages—which are the most demanding days of racing.
- Overall though, professional cyclists were able to obtain a reasonable amount of good-quality sleep while competing in the Tour de France (for men) and Tour de France Femmes (for women).
- This study demonstrates that it is now feasible to use validated fitness trackers to assess the night-time recovery of professional athletes during multiple-day endurance events.

**Supplementary Information:**

The online version contains supplementary material available at 10.1186/s40798-024-00716-6.

## Background

In professional men’s cycling, the most physically and mentally demanding events are the Grand Tours—i.e., Tour de France, Giro d’Italia and Vuelta a España [1]. The Grand Tours are held over three consecutive weeks of racing with daily stages covering a variety of terrain—flat, hilly and mountain. Typically, the Tour de France imposes the highest total exercise loads on its participants due to the duration and length of the daily stages—the longest by duration last up to 5 h and the longest by length cover up to 300 km [2, 3]. In recent decades, competitors in the Tour de France have covered an average distance of 3,650 ± 208 km, with the winners taking an average of 92 ± 6 h to complete the race [1]. There are no three-week tours in women’s professional cycling, but the most prestigious events, and potentially the most demanding, are the one-week tours that are ‘sister’ events to the men’s Grand Tours, known as Tour de France Femmes, Giro Donne and Vuelta Femenina.

The physiological demands of the Grand Tours are well above those that can be undertaken by most endurance-trained athletes [2]. To this end, the requirements of the main types of stages during Grand Tours have been well described in terms of both the volume and intensity of exercise [1–7]. For example, exercise duration is shortest for time trial stages and longest for flat and mountain stages; exercise intensity is low to moderate during flat stages, moderate to high during mountain stages, and high during time trial stages; and physiological load is greatest during mountain stages (average power output = 345–380 W) compared to flat stages (average power output = 200–250 W) [1, 4]. Understanding the physiological demands imposed on cyclists during Grand Tours is useful in terms of the design of training strategies to optimise potential performance [4], but this information does not provide insight into the capacity of cyclists to physiologically recover between stages.

Sleep is considered one of the major forms of recovery from exercise [8], but it can be disrupted following single bouts of ultra-endurance exercise [9]. Obtaining sufficient sleep may be particularly challenging during Grand Tours because cyclists must complete extreme bouts of endurance exercise each day with little time to recover before the next stage of racing. Specifically, road cyclists report needing 8.2 h of sleep per night to feel fully rested [10], but during one-week multi-stage races, they obtain an average of only ~ 7 h of sleep per night [11, 12]. Furthermore, in simulated Grand Tours, average sleep duration declines over consecutive weeks of racing from 7.4 h to 7.0 h [13]. In addition, indicators of recovery based on autonomic activity, i.e., resting heart rate and heart rate variability, also decline over consecutive weeks of simulated racing [14].

For cyclists competing in Grand Tours, insufficient recovery between stages because of poor sleep could affect performance during subsequent stages of the race. The physiological demands placed on cyclists during Grand Tours have been quantified, but the impact of these demands on daily recovery in terms of sleep and autonomic activity are yet to be determined. The emergence of wearable technologies now makes it possible to monitor sleep and autonomic activity non-invasively during competition [15]. In the present study, data were collected using one such wearable technology, i.e., WHOOP 4.0 (Whoop, Inc., Boston, MA, USA), with professional male and female cyclists competing in the 2022 editions of the Tour de France and Tour de France Femmes, respectively. The aims of the study were to (i) quantify the impact of race days on night-time sleep and autonomic activity in professional male and female cyclists; (ii) examine whether the characteristics of a race stage—i.e., rest, flat, hilly, mountain or time trial for males and flat, hilly or mountain for females—differentially affect night-time sleep and autonomic activity in professional male and female cyclists; and (iii) determine whether there is a cumulative effect of racing over consecutive weeks on night-time sleep and autonomic activity in professional male cyclists. It was hypothesised that night-time sleep and autonomic activity would be poorer following stages with relatively high physiological demands (i.e., mountain stages) compared to stages with relatively low physiological demands (i.e., flat stages).

## Methods

### Participants

Seventeen professional road cyclists from teams competing in the 2022 editions of the Tour de France and Tour de France Femmes provided informed consent for their data to be included in the present study. To be eligible for inclusion, cyclists had to be competing in the 2022 edition of the Tour de France or the Tour de France Femmes and had to volunteer to wear a fitness tracker (WHOOP 4.0) to monitor recovery during their respective race. These inclusion criteria limited the number of potential participants, but the sample size of the present study was similar to, or larger than, the sample sizes in other studies that have collected physiological data with professional cyclists during multiple-day races [5, 7, 11, 12, 14]. The male cyclists (*n* = 8; age: 28.0 ± 2.5 years; mass: 70.3 ± 4.6 kg; height: 181.6 ± 4.9 cm; mean ± 95% confidence interval) were members of the same team. The female cyclists (*n* = 9; age: 26.7 ± 3.1 years; mass: 58.0 ± 4.4 kg; height: 169.8 ± 5.6 cm; mean ± 95% confidence interval) were members of two teams (team 1: *n* = 4; team 2: *n* = 5). The cyclists were free to consume substances that may affect night-time sleep during the data collection period (e.g., alcohol, caffeine, medications, supplements, etc.), but information regarding such consumption was not collected. Informed consent was obtained from all participants included in the study. The procedure for data collection complied with the Declaration of Helsinki and was approved by CQUniversity’s Human Research Ethics Committee (0000022344; 21/04/2020).

### Race Schedule

#### Male Cyclists

In 2022, the Tour de France included 21 stages of racing from the 1st of July to the 24th of July (Table 1). The race consisted of two individual time trials (stages 1, 20), six flat stages (2, 3, 13, 15, 19, 21), seven hilly stages (4–6, 8, 10, 14, 16), six mountain stages (7, 9, 11, 12, 17, 18), two rest days, and one transfer day—with a total distance of 3,581 km and a total elevation gain of 47,026 m.

**Table 1:** 2022 Tour de France route and classification

Stage	Course	Distance (km)	Classification	Elevation (m)
1	Copenhagen > Copenhagen	13.2	Time Trial	22
2	Roskilde > Nyborg	202.5	Flat	1116
3	Vejle > Sønderborg	182.0	Flat	1335
Rest	Sønderborg > Dunkerque	–	–	–
4	Dunkerque > Calais	171.5	Hilly	1842
5	Lille Métropole > Arenberg Porte du Hainaut	157.0	Hilly	822
6	Binche > Longwy	220.0	Hilly	2503
7	Tomblaine > La Super Planche des Belles Filles	176.5	Mountain	2521
8	Dole > Lausanne	186.5	Hilly	2377
9	Aigle > Châtel Les Portes du Soleil	193.0	Mountain	2900
Rest	Châtel Les Portes du Soleil > Morzine Les Portes du Soleil	–	–	–
10	Morzine Les Portes du Soleil > Megève	148.5	Hilly	2296
11	Albertville > Col du Granon Serre Chevalier	152.0	Mountain	3993
12	Briançon > Alpe D’Huez	165.5	Mountain	4813
13	Le Bourg D’Oisans > Saint-Étienne	193.0	Flat	1782
14	Saint-Étienne > Mende	192.5	Hilly	3533
15	Rodez > Carcassonne	202.5	Flat	2272
Rest	Carcassonne	–	–	–
16	Carcassone > Foix	178.5	Hilly	3118
17	Saint-Gaudens > Peyragudes	130.0	Mountain	3338
18	Lourdes > Hautacam	143.5	Mountain	3994
19	Casteinau-Magnoac > Cahors	188.5	Flat	1315
20	Lacapelle-Marival > Rocamdour	40.7	Time Trial	383
21	Paris La Défense Arena > Paris Champs-Élysées	166.0	Flat	754

#### Female Cyclists

In 2022, the Tour de France Femmes included eight stages of racing from the 24th of July to the 31st of July (Table 2). The race did not have any time trials or rest days, but it consisted of three flat stages (1, 2, 5), three hilly stages (3, 4, 6), and two mountain stages (7, 8) —with a total distance of 1,033 km and a total elevation gain of 12,707 m.

**Table 2:** 2022 Tour de France Femmes route and classification

Stage	Course	Distance (km)	Classification	Elevation (m)
1	Tour Eiffel > Paris Champs-Élysées	81.6	Flat	421
2	Meaux > Provins	136.4	Flat	846
3	Reims > Épernay	133.5	Hilly	1587
4	Troyes > Bar-Sur-Aube	126.8	Hilly	1397
5	Bar-Le-Duc > Saint-Dié-Des-Vosges	175.6	Flat	1666
6	Saint-Dié-Des-Vosges > Rosheim	129.2	Hilly	1581
7	Sélestat > Le Markstein	127.1	Mountain	2738
8	Lure > La Super Planche Des Belles Filles	123.3	Mountain	2472

### Night-Time Sleep and Autonomic Activity

The cyclists’ sleep and autonomic activity were monitored using a fitness tracker worn on the wrist (WHOOP 4.0, Boston, MA, USA). For each night-time sleep opportunity, the following sleep variables were obtained: sleep onset time, sleep offset time, time in bed (time between sleep onset and sleep offset), total sleep time, sleep efficiency (total sleep time / time in bed × 100), light sleep (expressed as a percentage of time in bed), slow wave sleep (expressed as a percentage of time in bed), and rapid eye movement (REM) sleep (expressed as a percentage of time in bed). Resting heart rate (beats·min^−1^) was calculated as the mean heart rate of non-wake periods of the primary sleep episode. Heart rate variability (rMSSD, ms) was calculated using the root-mean-square of successive differences between heartbeats formula across the non-wake periods of the primary sleep episode. In all analyses, the sleep and autonomic activity associated with a particular stage are the data collected on the night immediately following that stage.

Previous generations of the WHOOP strap (WHOOP 2.0 and 3.0) have been validated against the gold standard for monitoring sleep (i.e., polysomnography) and cardiac activity (i.e., electrocardiogram) [15–17]. Compared with polysomnography, WHOOP 3.0’s agreement (and Cohen’s kappa) is 86% (κ = 0.44) for two-state categorisation of sleep periods (as sleep or wake) [15] and 60% (κ = 0.44) for multi-state categorisation of sleep periods (as a specific sleep stage or wake) [15]. Compared with electrocardiogram, WHOOP 3.0 underestimates resting heart rate by an average of 0.3 beats·min^−1^ and underestimates heart rate variability (rMSSD) by an average of 4.5 ms [15]. For both heart rate and heart rate variability, the intraclass correlations between WHOOP 3.0 and electrocardiogram are 0.99, i.e., excellent [15].

### Physiological Workload

A marker of the physiological workload undertaken during each race stage, i.e., training impulse (TRIMP), was estimated for each cyclist. Heart rate was measured using the WHOOP strap and a modified version of TRIMP was calculated using the following formula [18, 19]:

TRIMP = A·B·C.

where A is stage duration (minutes), B is [(HR_stage_—HR_rest_)/(HR_max_—HR_rest_)], and C equals 0.64·e^1.92B^. HR_stage_ is the average heart rate during the stage, HR_rest_ is resting heart rate (determined as the average of the resting heart rate values recorded during baseline), and HR_max_ is maximum heart rate (determined as the highest heart rate recorded during any race stage).

In addition to TRIMP, WHOOP ‘daily strain’ was calculated for each day of the study (including non-race days). WHOOP daily strain measures ‘total cardiovascular load’ on a proprietary, non-linear scale of 0 to 21 and is categorised into the following bands: light strain (0–9), moderate strain (10–13), high strain (14–17), and overreaching (18–21) [20].

## Statistical Analyses

The aims of the study were addressed by conducting a series of linear mixed effects models using the variance components covariance structure and restricted maximum likelihood estimation. Statistical analyses were performed using SPSS (version 28.0.0.0; IBM; Armonk, NY, USA). Results are reported as mean (± 95% confidence intervals) and were considered significant at *p* < 0.05. Separate linear mixed effects models were conducted for male and female cyclists as described in the following two subsections.

### Male Cyclists

Daily differences in TRIMP, daily strain, and night-time sleep and autonomic activity were examined by constructing separate models for each dependent variable with ‘day of race’ as a fixed effect and ‘participant’ as a random effect. For variables related to sleep and autonomic activity, ‘day of race’ included a pre-race baseline (i.e., Monday to Sunday at least one week prior to the first stage of the race), 21 race stages interspersed with 3 rest days, and a post-race period (i.e., days/nights 2–9 immediately after the race). For daily strain, ‘day of race’ included a pre-race baseline (as above), 19 stages interspersed with 3 rest days, and a post-race period (as above). For TRIMP, ‘day of race’ only included 19 stages because the timing and duration of specific exercise bouts during the pre-race baseline, rest days, and post-race period were not available. The two time trials (stages 1 and 20) were not included in the analyses for daily strain and TRIMP because the cyclists tended not to wear the WHOOP strap during these stages. If a main effect of ‘day of race’ was observed for daily strain or variables related to sleep and autonomic activity, pairwise comparisons with a Bonferroni correction were performed with ‘baseline’ as the reference category.

To examine whether the characteristics of a race stage affect TRIMP, daily strain, and night-time sleep and autonomic activity, separate models were constructed for each dependent variable with ‘stage classification’ as a fixed effect and ‘participant’ as a random effect. Stage classifications were based on the categorisations published by the race organisers, i.e., rest (rest days 1–3), flat (stages 2, 3, 13, 15, 19, 21), hilly (stages 4–6, 8, 10, 14, 16), mountain (stages 7, 9, 11, 12, 17, 18) and individual time trial (stages 1, 20). For variables related to sleep and autonomic activity, ‘stage classification’ included the categories of rest, flat, hilly, mountain and time trial. The last stage (stage 21) was not included in the flat category for these variables because they may be affected by additional factors associated with ending the race (e.g., travel commitments). For daily strain, ‘stage classification’ included the categories of rest, flat, hilly and mountain. For TRIMP, ‘stage classification’ included the categories of flat, hilly and mountain. The time trials (stages 1 and 20) were not included in the analyses for daily strain and TRIMP because the cyclists tended not to wear the WHOOP strap during these stages. Rest days were not included in the analysis for TRIMP because the timing and duration of specific exercise bouts on those days were not available. For all dependent variables, if a main effect of ‘stage classification’ was observed, all pairwise comparisons were performed with a Bonferroni correction applied.

To examine whether there is a cumulative effect of racing over consecutive weeks on TRIMP, daily strain, and night-time sleep and autonomic activity, separate models were constructed for each dependent variable with ‘week of race’ as a fixed effect and ‘participant’ as a random effect. For variables related to sleep and autonomic activity, ‘week of race’ included the categories of pre-race baseline (i.e., Monday to Sunday at least one week prior to the first stage of the race), week 1 (i.e., stages 1–9, excluding rest day 1), week 2 (i.e., stages 10–15, excluding rest day 2) and week 3 (i.e., stages 16–20, excluding rest day 3). The last stage (stage 21) was not included in the week 3 category for these variables because they may be affected by additional factors associated with ending the race. For daily strain, ‘week of race’ included the categories of pre-race baseline (as above), week 1 (i.e., stages 2–9, excluding rest day 1), week 2 (i.e., stages 10–15, excluding rest day 2) and week 3 (i.e., stages 16–19 and 21, excluding rest day 3). For TRIMP, ‘week of race’ was similar to that for daily strain, except that it did not include a pre-race baseline category because the timing and duration of specific exercise bouts on those days were not available. The time trials, i.e., stage 1 in week 1 and stage 20 in week 3, were not included in the analyses for daily strain and TRIMP because the cyclists tended not to wear the WHOOP strap during these stages. For all dependent variables, if a main effect of ‘week of race’ was observed, all pairwise comparisons were performed with a Bonferroni correction applied.

### Female Cyclists

Daily differences in TRIMP, daily strain, and night-time sleep and autonomic activity were examined by constructing separate models for each dependent variable with ‘day of race’ as a fixed effect and ‘participant’ as a random effect. For daily strain and variables related to sleep and autonomic activity, ‘day of race’ included a pre-race baseline (i.e., Monday to Sunday at least one week prior to the first day of the race), 8 race stages, and a post-race period (i.e., days/nights 2–9 immediately after the race). For TRIMP, ‘day of race’ only included 8 stages because the timing and duration of specific exercise bouts during the pre-race baseline and post-race period were not available. If a main effect of ‘day of race’ was observed for daily strain or variables related to sleep and autonomic activity, pairwise comparisons with a Bonferroni correction were performed with ‘baseline’ as the reference category.

To examine whether the characteristics of a race stage affect TRIMP, daily strain, and night-time sleep and autonomic activity, separate models were constructed for each dependent variable with ‘stage classification’ as a fixed effect and ‘participant’ as a random effect. Stage classifications were based on the categorisations published by the race organisers, i.e., flat (stages 1, 5), hilly (stages 2, 3, 6) and mountain (stages 7, 8). For all variables, ‘stage classification’ included flat, hilly and mountain, but the last stage (stage 8) was not included in the mountain category for variables related to night-time sleep and autonomic activity because they may be affected by additional factors associated with ending the race (e.g., travel commitments). For all dependent variables, if a main effect of ‘stage classification’ was observed, all pairwise comparisons were performed with a Bonferroni correction applied.

## Results

### Male Cyclists

#### Race Completion and Compliance (Fig. 1)

Of the eight male cyclists who participated in data collection, six completed all 21 stages of the race and two withdrew from the race. For the cyclists who withdrew, data collected prior to withdrawal were included in the analyses. Of the 56 nights available to calculate a baseline average, 55 nights were collected (98%); of the 166 nights available following successful completion of a stage of the race, 159 were collected (96%); and of the 56 nights available to calculate a post-race average, 48 were collected (86%).

**Fig. 1:**
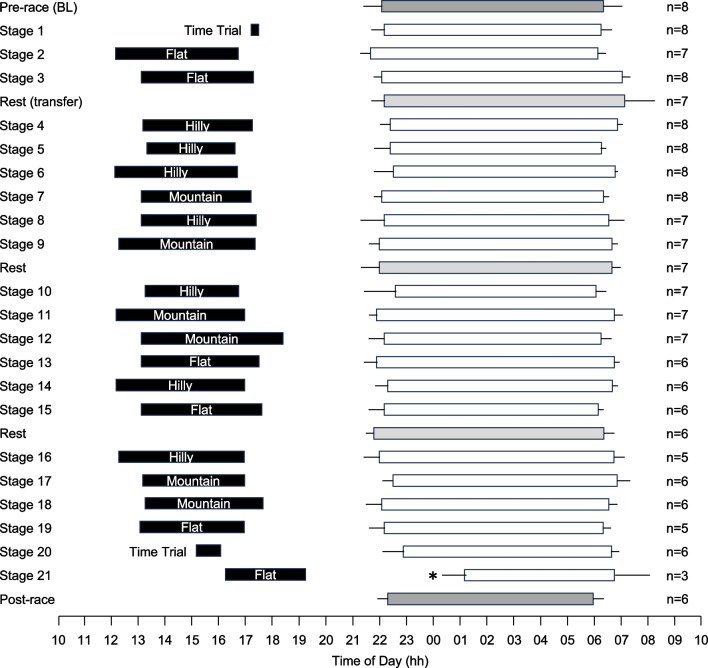
Timing of stages and sleep periods for male cyclists during the 2022 Tour de France. Each line represents a 24-h day from 10:00 AM to 10:00 AM. Black bars indicate the start/end times and duration of stages, based on the cyclists who participated in the study. The start/end times and duration of sleep periods for participating cyclists are represented by white bars for sleep after race stages, light grey bars for sleep after rest days and dark grey bars for sleep in the 14 days before and after competition. Data are mean and 95% confidence intervals. * indicates a significant difference from baseline (*p* < .05)

#### Baseline

During the pre-race baseline, the cyclists fell asleep at 22:03 h ± 40.8 min, woke at 06:21 h ± 43.2 min, spent 8.3 ± 0.2 h in bed, and obtained 7.2 ± 0.3 h of sleep with an efficiency of 87.0 ± 4.4%. The percentage of time in bed spent in each stage of sleep was 45.1 ± 5.5% for light sleep, 18.5 ± 2.5% for slow wave sleep and 23.4 ± 4.3% for REM sleep. In comparison, for male/female non-athletes of a similar age (25 years), the percentage of time in bed spent in each stage of sleep is 54.8% for light sleep, 16.3% for slow wave sleep and 20.7% for REM sleep [21].

For autonomic activity during the pre-race baseline, resting heart rate during sleep was 41.8 ± 4.5 beats·min^−1^ and heart rate variability during sleep was 108.5 ± 17.0 ms. In comparison, for male/female non-athletes of a similar age (20–29 years), resting heart rate during sleep is 54.2 beats·min^−1^ and heart rate variability during sleep is 82.9 ms [22].

#### Day of Race (Figs. 1, 2, 3, 4, 5; Additional file 1: Tables 1A, 2A, 3A, 4A)

**Fig. 2:**
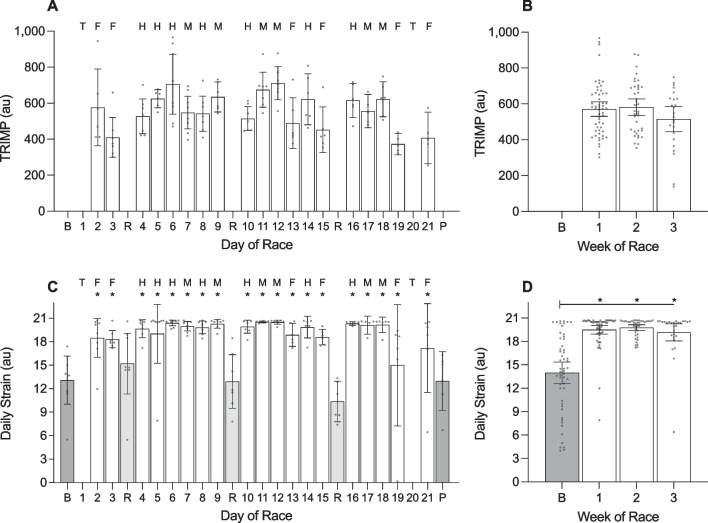
Training impulse (TRIMP) and daily strain of male cyclists during the Tour de France plotted as a function of day of race (panels **a** and **c**) and week of race (panels **b** and **d**). AU, arbitrary units. ‘B’ represents the baseline mean calculated at least one week prior to the first day of the race; ‘P’ indicates the post-race mean calculated from post-race night 2 to post-race night 9; ‘R’ indicates rest days; ‘T’ indicates individual time trial; ‘F’ indicates flat stages; ‘H’ indicates hilly stages; ‘M’ indicates mountain stages. Data are presented as mean (bars) and 95% confidence intervals (error bars). Closed circles represent individual cyclists. In the panel **a** for TRIMP, the results of the post-hoc analysis are not shown. In panels **c** and **d** for daily strain, * indicates a significant difference from baseline (*p* < .05)

**Fig. 3:**
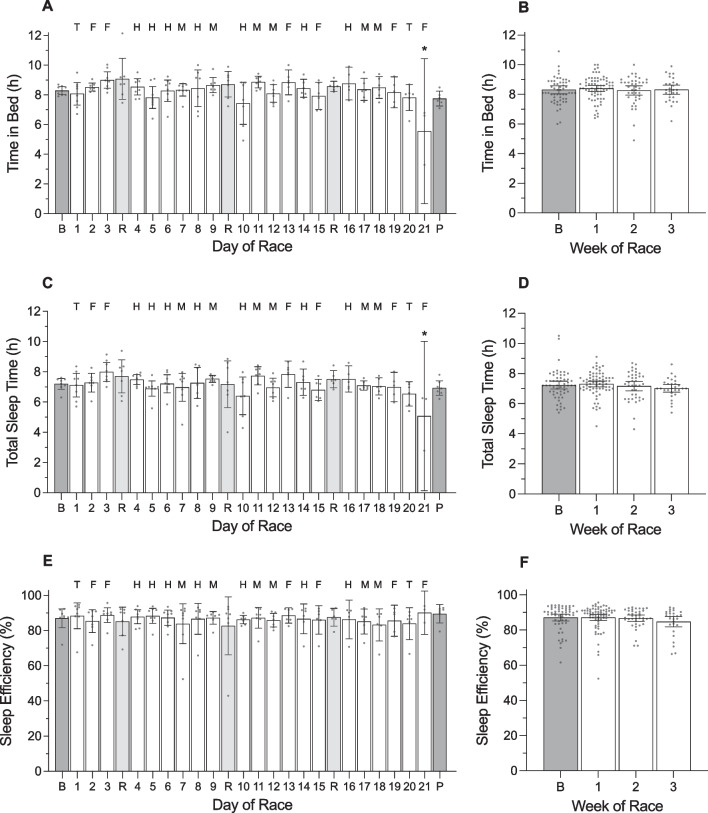
Time in bed, total sleep time, and sleep efficiency of male cyclists during the Tour de France plotted as a function of day of race (panels **a**, **c**, **e**) and week of race (panels **b**, **d**, **f**). ‘B’ represents the baseline mean calculated at least one week prior to the first day of the race; ‘P’ indicates the post-race mean calculated from post-race night 2 to post-race night 9; ‘R’ indicates rest days; ‘T’ indicates individual time trial; ‘F’ indicates flat stages; ‘H’ indicates hilly stages; ‘M’ indicates mountain stages. Data are presented as mean (bars) and 95% confidence intervals (error bars). Closed circles represent individual cyclists. In panels **a** and **c**, * indicates a significant difference from baseline (*p* < .05)

**Fig. 4:**
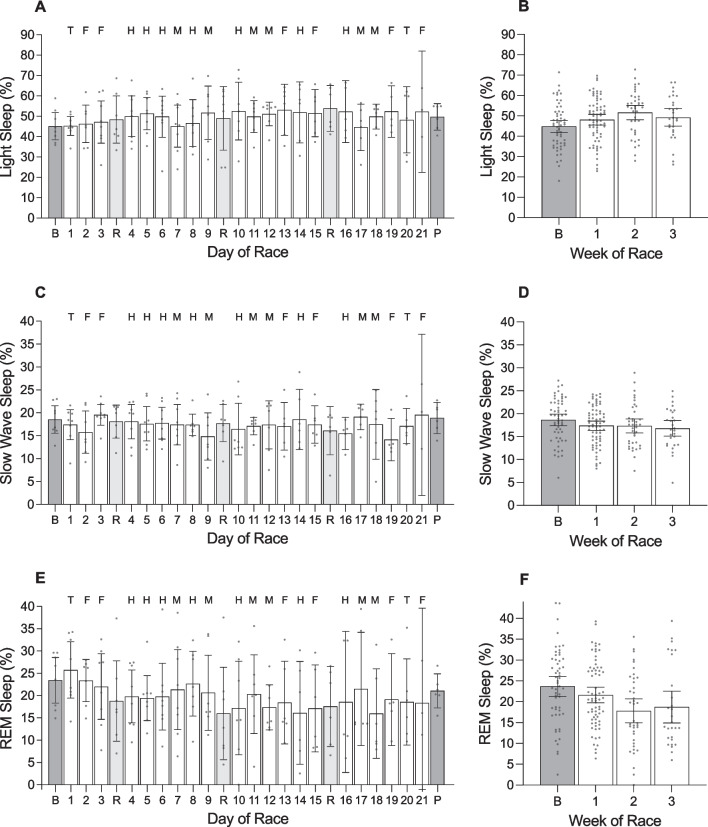
The percentage of light sleep, slow wave sleep and rapid eye movement (REM) sleep obtained by male cyclists during the Tour de France plotted as a function of day of race (panels **a**, **c**, **e**) and week of race (panels **b**, **d**, **f**). ‘B’ represents the baseline mean calculated at least one week prior to the first day of the race; ‘P’ indicates the post-race mean calculated from post-race night 2 to post-race night 9; ‘R’ indicates rest days; ‘T’ indicates individual time trial; ‘F’ indicates flat stages; ‘H’ indicates hilly stages; ‘M’ indicates mountain stages. Data are presented as mean (bars) and 95% confidence intervals (error bars). Closed circles represent individual cyclists

**Fig. 5:**
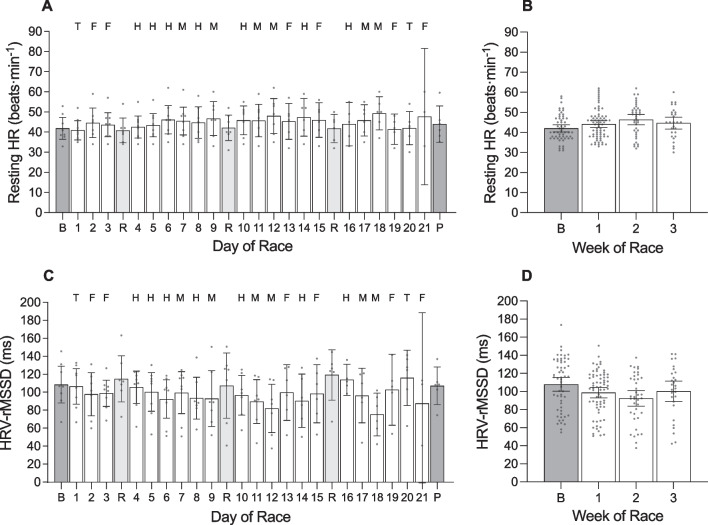
Resting heart rate (HR) and heart rate variability (HRV) of male cyclists during the Tour de France plotted as a function of day of race (panels **a** and **c**) and week of race (panels **b** and **d**). HRV was calculated using the root-mean-square of successive differences between heartbeats (rMSSD and is reported in milliseconds (ms). ‘B’ represents the baseline mean calculated at least one week prior to the first day of the race; ‘P’ indicates the post-race mean calculated from post-race night 2 to post-night 9; ‘R’ indicates rest days; ‘T’ indicates individual time trial; ‘F’ indicates flat stages; ‘H’ indicates hilly stages; ‘M’ indicates mountain stages. Data are presented as mean (bars) and 95% confidence intervals (error bars). Closed circles represent individual cyclists. In the left and right panels, * indicates a significant difference from baseline (*p* < .05)

There was a main effect of ‘day of race’ on TRIMP and daily strain (Table 3). TRIMP was highest during stage 6 (hilly) and stage 12 (mountain) and lowest during stages 3 and 19 (both flat). Daily strain was higher on all days of racing compared to baseline (stages 2–18: *p* =  < 0.001; stage 19: *p* = 0.007; stage 21 *p* = 0.008), but did not differ from baseline on rest days 1–3 (*p* = 0.999, *p* = 0.999, *p* = 0.518, respectively) or during the post-race period (*p* = 0.999).

**Table 3:** Results of the linear mixed models examining variables across days of the race in male cyclists

Variable	*F*	df	*p* value
TRIMP (au)	4.50	1, 18	< .001
Daily strain (au)	11.51	1, 23	< .001
Sleep onset (hh:mm)	2.49	1, 25	< .001
Sleep offset (hh:mm)	2.02	1, 25	.005
Time in bed (h)	2.83	1, 25	< .001
Total sleep time (h)	2.05	1, 25	.004
Sleep efficiency (%)	0.36	1, 25	.998
Light sleep (%)	0.39	1, 25	.996
Slow wave sleep (%)	0.52	1, 25	.972
REM sleep (%)	0.56	1, 25	.954
Resting HR (beats·min^−1^)	0.57	1, 25	.946
HRV-rMSSD (ms)	0.95	1, 25	.537

*TRIMP* training impulse, *au* arbitrary units, *REM* rapid eye movement, *HR* heart rate, *HRV* heart rate variability, *rMSSD* root mean square of successive differences, *ms* milliseconds

During the race, the cyclists fell asleep at 22:18 h ± 7.8 min, woke at 06:36 h ± 5.4 min, spent 8.4 ± 0.2 h in bed, and obtained 7.2 ± 0.1 h of sleep with an efficiency of 86.4 ± 1.2%. The percentage of time in bed spent in each stage of sleep was 49.5 ± 1.7% for light sleep, 17.3 ± 0.7% for slow wave sleep and 19.6 ± 1.4% for REM sleep. There was a main effect of ‘day of race’ on sleep onset time, sleep offset time, time in bed, and total sleep time (Table 3). Compared with baseline, the cyclists fell asleep later on the night of the final stage (+ 3.2 h; *p* < 0.001), spent less time in bed on the night of the final stage (− 2.7 h; *p* < 0.001), and obtained less sleep on the night of the final stage (− 2.1 h; *p* < 0.008). There was a main effect of ‘day of race’ on sleep offset time but none of the pairwise comparisons with baseline were significantly different.

For autonomic activity during the race, resting heart rate during sleep was 44.5 ± 1.2 beats·min^−1^ and heart rate variability during sleep was 99.1 ± 4.2 ms. There was no main effect of ‘day of race’ on resting heart rate during sleep or heart rate variability during sleep (Table 3).

#### Week of Race (Figs. 2, 3, 4, 5; Additional file 1: Table 5A)

There was no main effect of ‘week of race’ on TRIMP, but there was a main effect of ‘week of race’ on daily strain (Table 4). Compared with baseline, daily strain was higher during week 1 (+ 6.4 au; *p* < 0.001), week 2 (+ 6.6 au; *p* < 0.001), and week 3 (+ 6.4 au; *p* < 0.001). Daily strain was not different between week 1 and week 2 (*p* = 0.999), between week 1 and week 3 (*p* = 0.999), or between week 2 and week 3 (*p* = 0.999). There was no main effect of ‘week of race’ on any of the variables related to night-time sleep and autonomic activity (Table 4).

**Table 4:** Results of the linear mixed models examining variables across weeks of the race in male cyclists

Variable	*F*	df	*p* value
TRIMP (au)	2.85	1, 2	.062
Daily strain (au)	29.87	1, 3	< .001
Sleep onset (hh:mm)	0.50	1, 3	.684
Sleep offset (hh:mm)	1.46	1, 3	.228
Time in bed (h)	0.28	1, 3	.841
Total sleep time (h)	0.85	1, 3	.470
Sleep efficiency (%)	0.80	1, 3	.496
Light sleep (%)	1.28	1, 3	.285
Slow wave sleep (%)	0.34	1, 3	.800
REM sleep (%)	2.33	1, 3	.077
Resting HR (beats·min^−1^)	1.13	1, 3	.339
HRV-rMSSD (ms)	1.14	1, 3	.337

*TRIMP* training impulse, *au* arbitrary units, *REM* rapid eye movement, *HR* heart rate, *HRV* heart rate variability, *rMSSD* root mean square of successive differences, *ms* milliseconds

#### Stage Classification (Figs. 1, 6; Additional file 1: Table 6A)

**Fig. 6:**
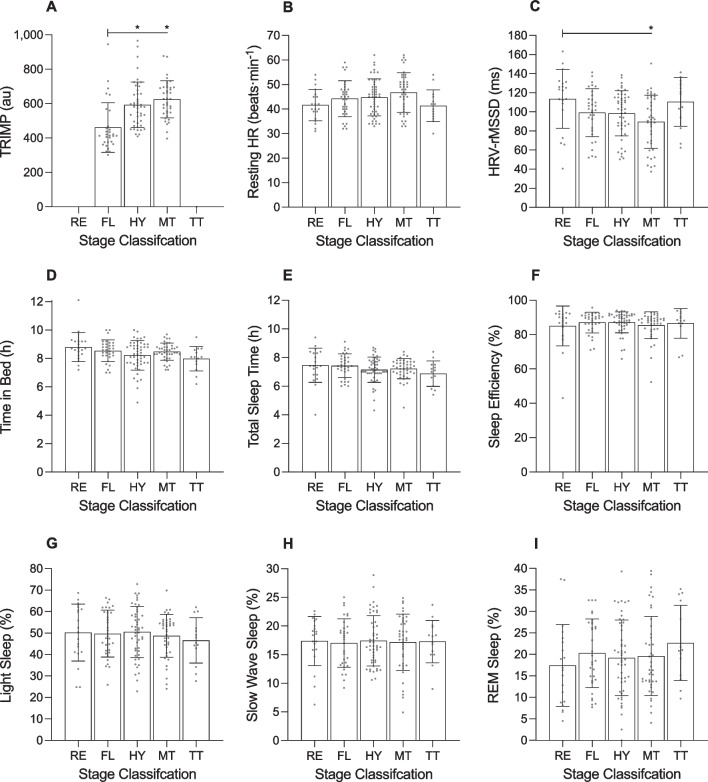
Training impulse (TRIMP; panel **a**), resting heart rate (HR; panel **b**), heart rate variability (HRV; panel **c**) and sleep (panels **d**–**i**) of male cyclists during the Tour de France plotted as a function of stage classification. AU, arbitrary units. HRV was calculated using the root-mean-square of successive differences between heartbeats (rMSSD and is reported in milliseconds (ms). ‘RE’ indicates transfer/rest days; ‘FL’ indicates flat stages; ‘HY’ indicates hilly stages; ‘MT’ indicates mountain stages; and ‘TT’ indicates time trials. Data are presented as mean (bars) and 95% confidence intervals (error bars). Closed circles represent individual cyclists. * in panels **a** and **c** indicates a significant difference between stage classifications (*p* < .05)

The cyclists covered 193.3 ± 2.7 km in 4.1 ± 0.2 h during flat stages, 179.5 ± 6.2 km in 4.2 ± 0.2 h during hilly stages, and 161.4 ± 6.4 km in 4.6 ± 0.2 h during mountain stages. There was a main effect of ‘stage classification’ on TRIMP and daily strain (Table 5). Compared with flat stages, TRIMP was higher for hilly stages (+ 139.0 au;

**Table 5:** Results of the linear mixed models examining variables across stage classifications in male cyclists

Variable	*F*	Df	*p* value
TRIMP (au)	12.60	1, 3	< .001
Daily strain (au)	70.58	1, 3	< .001
Sleep onset (hh:mm)	1.87	1, 4	.119
Sleep offset (hh:mm)	0.89	1, 4	.469
Time in bed (h)	2.67	1, 4	.034
Total sleep time (h)	1.46	1, 4	.217
Sleep efficiency (%)	0.47	1, 4	.756
Light sleep (%)	0.42	1, 4	.797
Slow wave sleep (%)	0.05	1, 4	.996
REM sleep (%)	0.79	1, 4	.531
Resting HR (beats·min^−1^)	2.38	1, 4	.054
HRV-rMSSD (ms)	3.53	1, 4	.009

*TRIMP* training impulse, *au* arbitrary units, *REM* rapid eye movement, *HR* heart rate, *HRV* heart rate variability, *rMSSD* root mean square of successive differences, *ms* milliseconds

*p* < 0.001) and higher for mountain stages (+ 171.9 au; *p* < 0.001), but did not differ between hilly stages and mountain stages (*p* = 0.240). Compared with rest days, daily strain was higher for flat stages (+ 5.9 au; *p* < 0.001), hilly stages (+ 7.1 au; *p* < 0.001), and mountain stages (+ 7.6 au; *p* < 0.001). Compared with flat stages, daily strain was higher for hilly stages (+ 1.2 au; *p* = 0.049) and mountain stages (+ 1.6 au; *p* = 0.004). There was a main effect of ‘stage classification’ on time in bed and heart rate variability during sleep (Table 5). Time in bed was shortest following the individual time trials compared to the other stage classifications, but none of the pairwise comparisons were significantly different. Heart rate variability during sleep was lower following mountain stages than following rest days (− 23.9 ms; *p* = 0.010), but none of the other pairwise comparisons were significantly different.

### Female Cyclists

#### Race Completion and Compliance (Fig. 7)

**Fig. 7:**
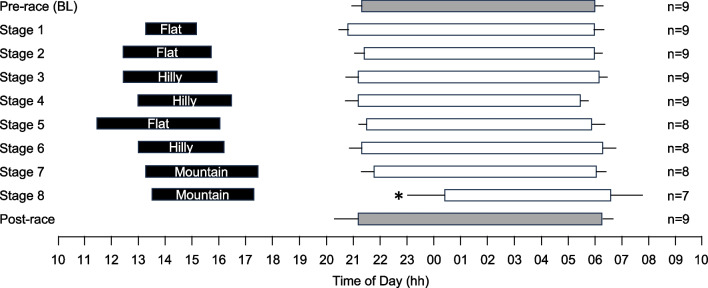
Timing of stages and sleep periods for female cyclists during the 2022 Tour de France Femmes. Each line represents a 24-h day from 10:00 AM to 10:00 AM. Black bars indicate the start/end times and duration of stages, based on the cyclists who participated in the study. The start/end times and duration of sleep periods for participating cyclists are represented by white bars for sleep after race stages and dark grey bars for sleep in the 14 days before and after competition. Data are mean and 95% confidence intervals. * indicates a significant difference from baseline (*p* < .05)

Of the nine female cyclists who participated in data collection, eight completed all eight stages of the race and one withdrew from the race. For the cyclist who withdrew, data collected prior to withdrawal were included in the analyses. Of the 63 nights available to calculate a baseline average, 63 were collected (100%); of the 70 nights available following successful completion of a stage of the race, 66 were collected (94%); and of the 63 nights available to calculate a post-race average, 61 were collected (97%).

#### Baseline

During the pre-race baseline, the cyclists fell asleep at 21:18 h ± 26.4 min, woke at 05:58 h ± 20.4 min, spent 8.7 ± 0.4 h in bed, and obtained 7.7 ± 0.3 h of sleep with an efficiency of 88.8 ± 2.6%. The percentage of time in bed spent in each stage of sleep was 41.9 ± 3.6% for light sleep, 20.2 ± 1.8% for slow wave sleep and 26.7 ± 4.0% for REM sleep. In comparison, for male/female non-athletes of a similar age (25 years), the percentage of time in bed spent in each stage of sleep is 54.8% for light sleep, 16.3% for slow wave sleep and 20.7% for REM sleep [21].

For autonomic activity during the pre-race baseline, resting heart rate during sleep was 45.8 ± 4.9 beats·min^−1^ and heart rate variability during sleep was 119.8 ± 26.4 ms. In comparison, male/female non-athletes of a similar age (20–29 years) have resting heart rate during sleep of 54.2 beats·min^−1^ and heart rate variability during sleep of 82.9 ms [22].

#### Day of race (Figs. 7, 8, 9, 10, 11; Additional file 1: Tables 7A, 8A, 9A, 10A)

**Fig. 8:**
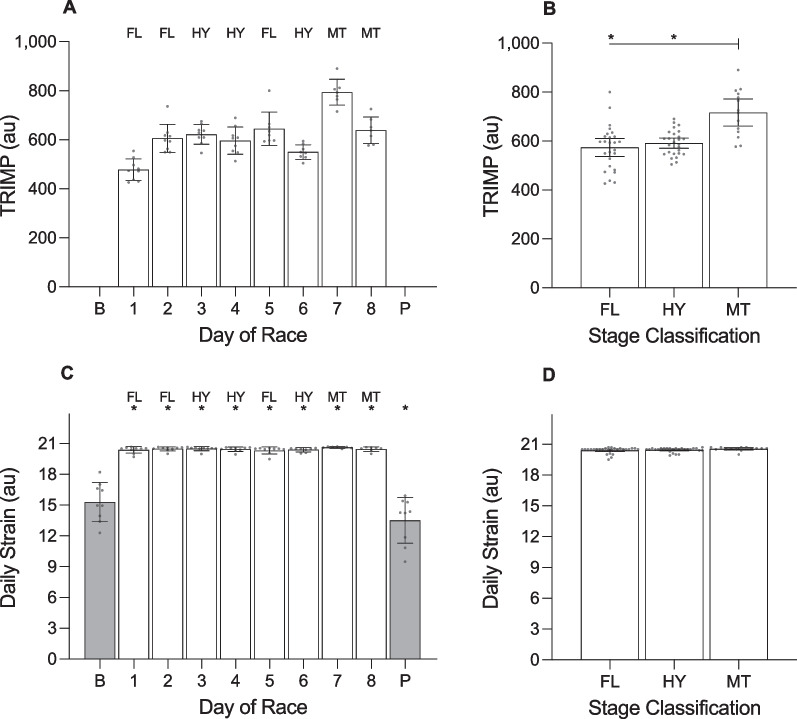
Training impulse (TRIMP) and daily strain of female cyclists during the Tour de France Femmes plotted as a function of day of race (panels **a** and **c**) and stage classification (panels **b** and **d**). AU, arbitrary units. ‘B’ represents the baseline mean calculated at least one week prior to the first day of the race; ‘P’ indicates the post-race mean calculated from post-race night 2 to post-race night 9; ‘FL’ indicates flat stages; ‘HY’ indicates hilly stages; ‘MT’ indicates mountain stages. Data are presented as mean (bars) and 95% confidence intervals (error bars). Closed circles represent individual cyclists. In panel **d**, * indicates a significant difference from baseline (*p* < .05)

**Fig. 9:**
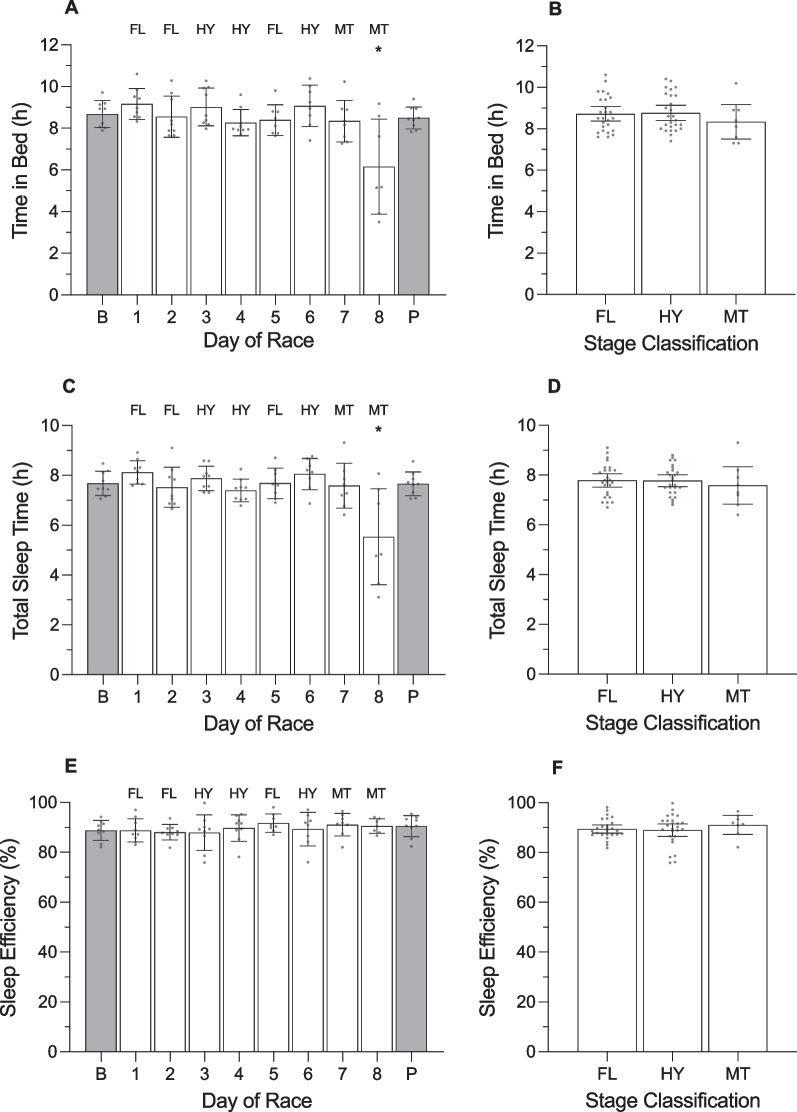
Time in bed, total sleep time, and sleep efficiency of female cyclists during the Tour de France Femmes plotted as a function of day of race (panels **a**, **c**, **e**) and stage classification (panels **b**, **d**, **f**). ‘B’ represents the baseline mean calculated at least one week prior to the first day of the race; ‘P’ indicates the post-race mean calculated from post-race night 2 to post-race night 9; ‘FL’ indicates flat stages; ‘HY’ indicates hilly stages; ‘MT’ indicates mountain stages. Data are presented as mean (bars) and 95% confidence intervals (error bars). Closed circles represent individual cyclists. In panels **a** and **d**, * indicates a significant difference from baseline (*p* < .05)

**Fig. 10:**
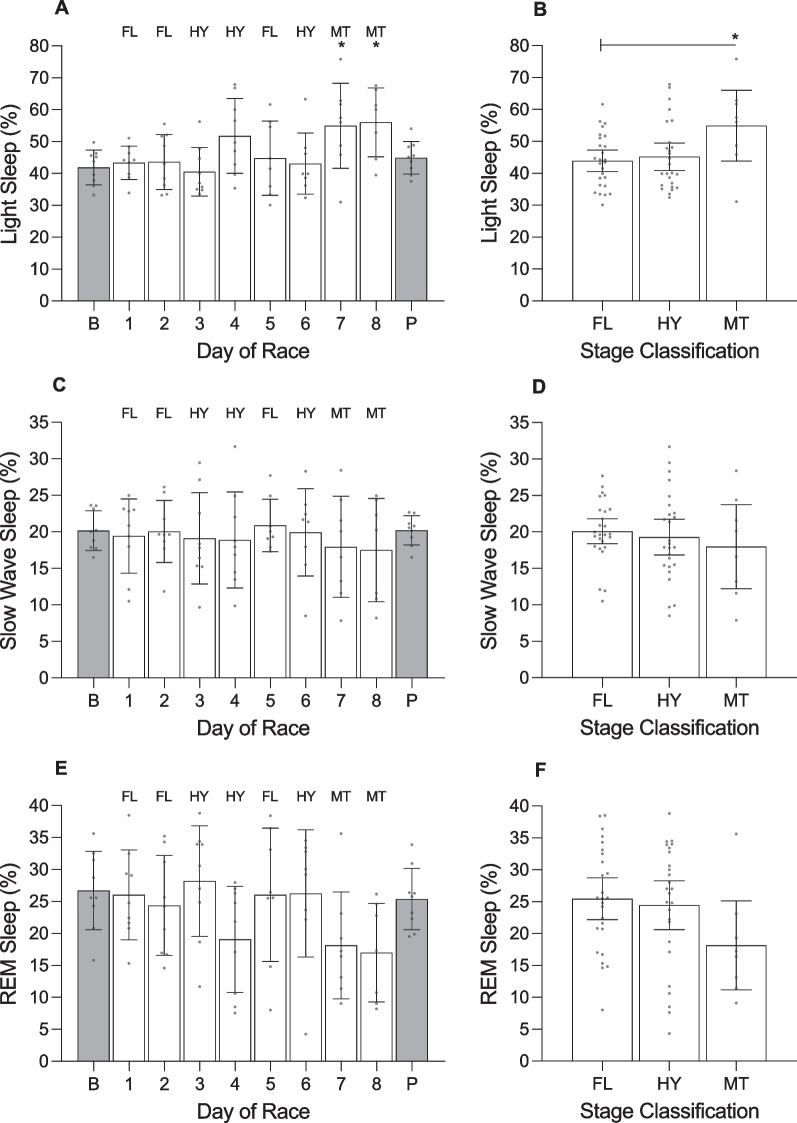
The percentage of light sleep, slow wave sleep and rapid eye movement (REM) sleep obtained by female cyclists during the Tour de France Femmes plotted as a function of day of race (panels **a**, **c**, **e**) and stage classification (panels **b**, **d**, **f**). ‘B’ represents the baseline mean calculated at least one week prior to the first day of the race; ‘P’ indicates the post-race mean calculated from post-race night 2 to post-race night 9; ‘FL’ indicates flat stages; ‘HY’ indicates hilly stages; ‘MT’ indicates mountain stages. Data are presented as mean (bars) and 95% confidence intervals (error bars). Closed circles represent individual cyclists. In panel **a**, * indicates a significant difference from baseline (*p* < .05). In panel **b** * indicates a significant difference between stage classifications (*p* < .05)

**Fig. 11:**
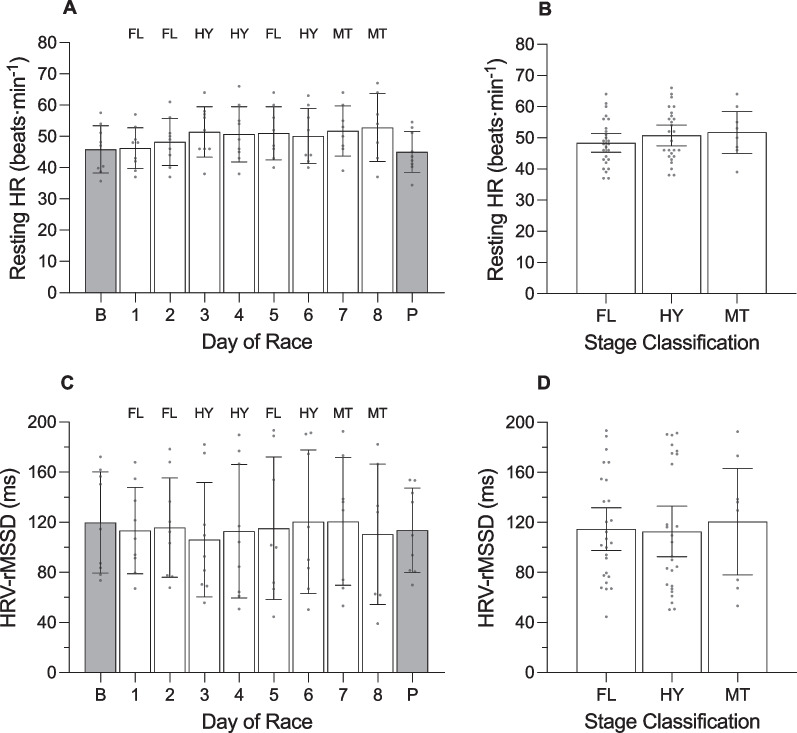
Resting heart rate (HR) and heart rate variability (HRV) of female cyclists during the Tour de France Femmes plotted as a function of day of race (panels **a** and **c**) and stage classification (panels **b** and **d**). HRV was calculated using the root-mean-square of successive differences between heartbeats (rMSSD and is reported in milliseconds (ms). ‘B’ represents the baseline mean calculated at least one week prior to the first day of the race; ‘P’ indicates the post-race mean calculated from post-race night 2 to post-race night 9; ‘FL’ indicates flat stages; ‘HY’ indicates hilly stages; ‘MT’ indicates mountain stages. Data are presented as mean (bars) and 95% confidence intervals (error bars). Closed circles represent individual cyclists

There was a main effect of ‘day of race’ on TRIMP and daily strain (Table 6). TRIMP was highest during stage 7 (mountain) and lowest during stage 1 (flat). Compared with baseline, daily strain was higher on stage 1 (+ 5.1 au;

**Table 6:** Results of the linear mixed models examining variables across days of the race in female cyclists

Variable	*F*	df	*p* value
TRIMP (au)	24.05	1, 7	< .001
Daily strain (au)	61.70	1, 9	< .001
Sleep onset (hh:mm)	10.01	1, 9	< .001
Sleep offset (hh:mm)	1.47	1, 9	.176
Time in bed (h)	5.26	1, 9	< .001
Total sleep time (h)	6.19	1, 9	< .001
Sleep efficiency (%)	0.60	1, 9	.795
Light sleep (%)	2.96	1, 9	.005
Slow wave sleep (%)	0.32	1, 9	.968
REM sleep (%)	2.07	1, 9	.043
Resting HR (beats·min^−1^)	0.98	1, 9	.461
HRV-rMSSD (ms)	0.08	1, 9	.999

*TRIMP* training impulse, *au* arbitrary units, *REM* rapid eye movement, *HR* heart rate, *HRV* heart rate variability, *rMSSD* root mean square of successive differences, *ms* milliseconds

*p* < 0.001), stage 2 (+ 5.2 au; *p* < 0.001), stage 3 (+ 5.2 au; *p* < 0.001), stage 4 (+ 5.1 au; *p* < 0.001), stage 5 (+ 5.1 au; *p* < 0.001), stage 6 (+ 5.1 au; *p* < 0.001), stage 7 (+ 5.3 au; *p* < 0.001), and stage 8 (+ 5.2 au; *p* < 0.001) and lower during the post-race period (− 1.8 au; *p* = 0.002).

During the race, the cyclists fell asleep at 21:37 h ± 18.6 min, woke at 06:03 h ± 10.8 min, spent 8.4 ± 0.3 h in bed, and obtained 7.5 ± 0.3 h of sleep with an efficiency of 89.6 ± 1.2%. The percentage of time in bed spent in each stage of sleep was 47.0 ± 2.6% for light sleep, 19.3 ± 1.3% for slow wave sleep and 23.3 ± 2.2% for REM sleep. There was a main effect of ‘day of race’ on sleep onset, time in bed, total sleep time, percentage of light sleep, and percentage of REM sleep (Table 6). Time in bed and total sleep time gradually decreased over the course of the race and the percentage of light sleep increased over the course of the race. Specifically, the cyclists fell asleep later (+ 3.1 h; *p* < 0.001), spent less time in bed (-2.5 h; *p* < 0.001), and obtained less sleep (-2.2 h; *p* < 0.001) on the night after completing stage 8 compared with baseline; and the percentage of light sleep obtained was higher following stage 7 (+ 13.0%; *p* = 0.043) and stage 8 (+ 14.1%; *p* = 0.030) compared with baseline. The percentage of REM sleep declined over the course of the race, but none of the pairwise comparisons with baseline were significantly different.

For autonomic activity during the race, resting heart rate during sleep was 50.2 ± 2.0 beats·min^−1^ and heart rate variability during sleep was 114.3 ± 11.2 ms. There was no main effect of ‘day of race’ on resting heart rate during sleep or heart rate variability during sleep (Table 6).

#### Stage Classification (Figs. 7, 8, 9, 10, 11; Additional file 1: Table 11A)

The cyclists covered 131.2 ± 14.8 km in 3.3 ± 0.4 h during flat stages, 129.7 ± 1.2 km in 3.4 ± 0.1 h during hilly stages, and 125.1 ± 1.0 km in 4.0 ± 0.1 h during the mountain stages. There was a main effect of ‘stage classification’ on TRIMP but no main effect of ‘stage classification’ on daily strain (Table 7). TRIMP was higher for mountain stages compared with flat stages (+ 142.9 au; *p* = 0.013) and compared with hilly stages (+ 125.3 au; *p* < 0.001), but did not differ between hilly stages and flat stages (*p* = 0.999). There was no main effect of ‘stage classification’ on most of the variables related to night-time sleep and autonomic activity, except for the percentage of light sleep obtained (Table 7). The percentage of light sleep was higher following the mountain stage compared with flat stages (+ 11.4%; *p* = 0.036), but did not differ between the mountain stage and hilly stages (*p* = 0.062) or between hilly stages and flat stages (*p* = 0.999).

**Table 7:** Results of the linear mixed models examining variables across stage classifications in female cyclists

Variable	*F*	df	*p* value
TRIMP (au)	16.31	1, 2	< .001
Daily strain (au)	1.36	1, 2	.265
Sleep onset (hh:mm)	2.04	1, 2	.140
Sleep offset (hh:mm)	0.14	1, 2	.873
Time in bed (h)	0.74	1, 2	.483
Total sleep time (h)	0.30	1, 2	.739
Sleep efficiency (%)	0.51	1, 2	.601
Light sleep (%)	3.76	1, 2	.029
Slow wave sleep (%)	0.48	1, 2	.619
REM sleep (%)	2.17	1, 2	.124
Resting HR (beats·min^−1^)	0.86	1, 2	.430
HRV-rMSSD (ms)	0.08	1, 2	.919

*TRIMP* training impulse, *au* arbitrary units, *REM* rapid eye movement, *HR* heart rate, *HRV* heart rate variability, *rMSSD* root mean square of successive differences, *ms* milliseconds

## Discussion

The major findings are that (i) the amount of sleep obtained by male and female cyclists did not change over the course of their respective races nor did autonomic activity during sleep; (ii) sleep quality was poorer after a mountain stage compared to flat stages in female cyclists; (iii) heart rate variability was lower after mountain stages compared to flat stages in male cyclists; and (iv) there was no cumulative effect of racing on sleep or autonomic activity during sleep in male cyclists.

There is some evidence to indicate that sleep is reduced on nights before and after competition [23–26] and that endurance exercise can also disrupt subsequent sleep [9]. In this regard, the Tour de France and the Tour de France Femmes are unique events because cyclists complete prolonged bouts of endurance exercise over successive days of competition. Surprisingly perhaps, total sleep time in the present study was not influenced by consecutive days of racing and remained relatively stable across most nights of the race. Specifically, male cyclists obtained an average of 7.2 h of sleep each night during their race compared to 7.2 h during the pre-race baseline and female cyclists obtained an average 7.5 h of sleep each night during their race compared to 7.7 h during the pre-race baseline. Interestingly, before and during their respective races, these cyclists obtained more sleep than the 6.3 h per night that other road cyclists obtain during a normal phase of training [10]. There are various possible explanations for the relatively good sleep observed in the present study, but the importance, and daily scheduling, of the events are probably the most critical. The Tour de France and Tour de France Femmes are the peak events in world cycling, so it is very likely that the cyclists prioritised sleep over other activities that tend to compete with the time available for sleep, in an attempt to obtain optimal recovery at night and achieve optimal performance during the day [27]. In addition, the schedule of racing may have been favourable in terms of cyclists’ opportunities for sleep. During training, athletes spend longer in bed and obtain more sleep when training starts in the mid-late morning compared with the early morning [28]. In this study, the average start time of race stages was 13:00 h for both male and female cyclists—the earliest start time for a stage of the Tour de France was 12:10 h and the earliest start time of a stage of the Tour de France Femmes was 11:45 h. With these start times, the average wake-up time on most days was 0600–0700 h for male cyclists and ~ 0600 h for female cyclists, which allowed them to spend a reasonable amount of time in bed and obtain a reasonable amount of sleep.

The Tour de France Femmes was a one-week race that started with less-demanding flat stages and ended with more-demanding mountain stages. Over the course of the race, the female cyclists exhibited poorer sleep quality, such that the percentage of light sleep was higher (+ 13.6%), and the percentage of REM sleep was lower (-9.2%), following the last two stages (both mountain stages) compared to baseline. Similar changes in light sleep and REM sleep have been observed following a single bout of ultra-endurance exercise in male athletes. For example, when compared to a day when no exercise was performed, light sleep increased (+ 27 min) and REM sleep decreased (-45 min) on the night following an ultra-triathlon [9]; and REM sleep was reduced (-6%) in well-trained cyclists following a single stage race (120–150 km) compared to a non-training/non-racing recovery period [29]. Prolonged endurance exercise can elevate core body temperature [29, 30], elicit muscle soreness [31, 32], and increase sympathetic activity [27]—all of which have the potential to disrupt sleep [9]. The probability that an increase in core body temperature is responsible for the changes in sleep observed in the present study may be small given that sleep opportunities began on average ~ 5.5 h after stage completion, over which time core body temperature should progressively decrease [29, 33]. An increase in muscle soreness or an increase in sympathetic activity may provide more likely explanations for an increase in light sleep and a decrease in REM sleep. For example, pain increases the number of arousals during sleep and the sleep arousal threshold to pain is lowest during light sleep compared to slow wave sleep or REM sleep [34]; and catecholamine excretion during the night increases following prolonged endurance exercise [29, 35], which may suppress REM sleep [28, 34]. In future, it may be useful to measure muscle soreness or catecholamine excretion following stages to determine the extent to which these factors disrupt sleep.

Indices of heart rate and heart rate variability are tools that can be used to quantify the acute stress-recovery responses of the autonomic nervous system following a bout of exercise [36, 37]. In the present study, heart rate variability was lower after mountain stages than after rest days in male cyclists. A similar negative relationship between heart rate variability and physiological load has been observed in male cyclists competing in the Vuelta a España [7] and in female cyclists riding the route of the 2017 Tour de France [14]. In each of these studies, heart rate variability was lowest following either the most demanding stages (i.e., mountain) [14] or after periods of racing in which physiological load was very high [7]. Despite an acute reduction in heart rate variability following mountain stages in male cyclists, there was no cumulative effect of racing on heart rate variability in male or female cyclists. For male cyclists, the presence of rest days and the distribution of flat stages between hilly or mountain stages during the race could provide sufficient time over which to recover and prevent further reductions in heart rate variability. For female cyclists, shorter race duration and placement of mountain stages at the end of the race could explain why heart rate variability remained stable throughout the race.

In the present study, the primary analyses focussed on the impact of the race schedule (i.e., consecutive days of racing over flat, hilly, mountain stages) on subsequent recovery (i.e., sleep and heart rate variability). Team strategy is another major factor that could affect the physiological and psychological demands placed on riders during the race and their subsequent recovery following each stage. Teams competing in the Tour de France or Tour de France Femmes consist of eight and six riders, respectively—a team leader, stage specialists (i.e., sprinters, climbers), and other support riders (i.e., domestiques). For each stage, riders may perform different roles depending on the performance of the team and the strategy of the team. For example, riders may be required to (i) set the pace at the front of the peloton to protect and conserve the energy of their team leader; (ii) defend their team leader against attacks from other riders; (iii) assist their team leader in initiating attacks against other riders to gain time or points; or (iv) form a breakaway to challenge for a stage win or support a teammate aiming for a stage win. These roles are likely to influence how well cyclists recover during the race. For example, heart rate variability was substantially lower for a domestique after the first 15 stages in the Tour of Spain compared to that of the team leader [7]. In the present study, there was insufficient data to examine the potential impact of individual and team strategies on subsequent recovery following each stage of the race. In future, it may be possible to accumulate data from multiple teams over multiple races to determine whether team strategy or team performance during a race affects subsequent recovery in terms of sleep and heart rate variability.

A major strength of the present study is that the data were collected with professional athletes while participating in a peak-level competition. However, there are some limitations that should be considered when interpreting the results. First, it is impractical to use the gold-standard equipment for assessing sleep, i.e., polysomnography, with professional athletes during peak events such as the Tour de France and Tour de France Femmes, so alternative equipment must be employed. In this study, a wearable fitness tracker, i.e., WHOOP 4.0, was used to assess night-time sleep and autonomic activity. An earlier version of the device, i.e., WHOOP 3.0, has excellent agreement with gold-standard measures of heart rate and heart rate variability and moderate agreement with gold-standard measures of sleep [15]. As with other fitness trackers that assess sleep, WHOOP is very good at detecting sleep in general, but its capacity to identify wake that occurs within a sleep period, and its capacity to identify particular stages of sleep, could be further improved [15]. These points should be considered when interpreting the results regarding sleep reported in the present study. Despite this caution regarding interpretation, this study demonstrates that it is now feasible to assess recovery metrics in professional athletes during multiple-day endurance events using validated fitness trackers. Second, compliance was very high on almost all nights before and after the race, but it was poorest on the night of the final stage of the race. For this reason, data on the night of the final stage was excluded from most analyses. For the male cyclists, this resulted in data being excluded from the final flat stage and for the female cyclists, this resulted in data being excluded from the final mountain stage. It is possible that exclusion of these data from some of the analyses may have altered the results. However, sleep/wake behaviours on the final night of racing may be influenced by factors other than the race (e.g., celebrations, media commitments, social demands, etc.) and as such, may not accurately reflect recovery. Finally, some aspects of the cyclists’ behaviour that could influence recovery were not captured during the data collection period. For example, napping is an effective strategy when athletes’ night-time sleep is restricted [38] and some athletes do nap during competition [39, 40], but naps were not included in the analyses in the present study. In addition, information regarding the cyclists’ nutritional intake and the use of alcohol, caffeine, supplements, and medications was not collected. In future studies of this nature, it may be possible to collect this information using the ‘journal’ function in the WHOOP device.

## Conclusion

The results of the present study indicate that some aspects of recovery were compromised in cyclists after the most demanding days of racing, i.e., mountain stages. Overall however, the cyclists obtained a reasonable amount of good-quality sleep while competing in these highly demanding endurance events. This study demonstrates that it is now feasible to assess recovery metrics in professional athletes during multiple-day endurance events using validated fitness trackers.

### Supplementary Information

**Additional file 1.** Tables of data (mean ± SD) corresponding to each of the figures presented in the manuscript.

## Data Availability

All data generated or analysed during the study are included in this published article [and in the supplementary material file].

## Abbreviations

AU: Arbitrary units
HR: Heart rate
HRV: Heart rate variability
ms: Milliseconds
PSG: Polysomnography
REM: Rapid eye movement
rMSSD: Root mean square of successive differences
TRIMP: Training impulse

## Declarations

### Preprint

The preprint version of this work is available on medRxiv https://www.medrxiv.org/content/10.1101/2023.07.06.23292280v2
