# Supplementary material for: The Night-Time Sleep and Autonomic Activity of Male and Female Professional Road Cyclists Competing in the Tour de France and Tour de France Femmes
Source: Sports Med Open. 2024 Apr 16;10:39. doi: 10.1186/s40798-024-00716-6 (PMC11021391; doi:10.1186/s40798-024-00716-6)
Supplement: Supplementary file 1 — Additional file 1. Tables of data (mean ± SD) corresponding to each of the figures presented in the manuscript. [file 40798_2024_716_MOESM1_ESM.pdf]

The night-time sleep and autonomic activity of male and female professional road cyclists competing in the Tour de France and Tour de France Femmes.

Sports Medicine – Open

Charli Sargent[1]·Summer Jasinski[2]·Emily R. Capodilupo[2]·Jeremy Powers[2]·Dean J. Miller[1]·Gregory D. Roach[1]

[1] CQUniversity, Appleton Institute for Behavioural Science, Adelaide, South Australia, Australia

[2] Whoop Inc., Data Science and Research, Boston, Massachusetts, United States of America

Corresponding Author:

Charli Sargent – charli.sargent@cqu.edu.au

Table 1A TRIMP and Daily Strain for baseline, each stage of the race, and post-race for male cyclists competing in the 2022 Tour de France

Day/Stage	Classification	TRIMP (au)	Daily Strain (au)
Baseline	-	-	13.1 ± 3.7 (n=8)
1	Time Trial	-	-
2	Flat	576.6 ± 203.5 (n=6)	18.5 ± 2.9 (n=8)
3	Flat	410.8 ± 119.8 (n=7)	18.3 ± 1.2 (n=7)
-	Rest	-	15.2 ± 4.6 (n=8)
4	Hilly	527.4 ± 104.6 (n=7)	19.7 ± 1.2 (n=7)
5	Hilly	624.9 ± 48.3 (n=6)	19.0 ± 4.5 (n=8)
6	Hilly	704.7 ± 198.7 (n=8)	20.4 ± 0.4 (n=8)
7	Mountain	547.8 ± 107.8 (n=8)	20.0 ± 0.7 (n=8)
8	Hilly	541.9 ± 105.4 (n=7)	19.8 ± 0.9 (n=7)
9	Mountain	634.7 ± 79.3 (n=6)	20.3 ± 0.6 (n=7)
-	Rest	-	12.9 ± 3.7 (n=7)
10	Hilly	514.8 ± 71.2 (n=7)	19.9 ± 0.9 (n=7)
11	Mountain	674.8 ± 105.0 (n=7)	20.5 ± 0.1 (n=7)
12	Mountain	710.9 ± 98.6 (n=7)	20.5 ± 0.3 (n=7)
13	Flat	489.4 ± 134.0 (n=6)	18.9 ± 1.4 (n=6)
14	Hilly	622.1 ± 135.0 (n=6)	19.9 ± 1.3 (n=6)
15	Flat	452.4 ±121.2 (n=6)	18.6 ± 1.0 (n=6)
-	Rest	-	10.4 ± 2.5(n=6)
16	Hilly	615.1 ± 90.3 (n=6)	20.3 ± 0.2 (n=6)
17	Mountain	556.3 ± 74.4 (n=5)	20.1 ± 0.9 (n=5)
18	Mountain	622.1 ± 92.7 (n=6)	20.2 ± 0.9 (n=6)
19	Flat	373.0 ± 47.6 (n=5)	15.0 ± 7.4 (n=6)
20	Time Trial	-	-
21	Flat	407.0 ± 115.4 (n=5)	17.2 ± 5.4 (n=6)
Post-race	-	-	13.0 ± 3.6 (n=6)

Data are mean ± standard deviation

n = the number of participants

TRIMP = training impulse, au = arbitrary units

Table 2A Sleep onset, sleep offset, time in bed and total sleep time for baseline, each stage of the race, and post-race for male cyclists competing in the 2022 Tour de France

Day/Stage	Classification	Cyclists (n)	Sleep Onset (hh:mm)	Sleep Offset (hh:mm)	TIB (h)	TST (h)
Baseline	-	8	22:03 ± 00:59	06:21 ± 01:02	8.3 ± 0.3	7.2 ± 0.4
1	Time Trial	8	22:14 ± 00:41	06:19 ± 00:37	8.1 ± 0.9	7.1 ± 0.9
2	Flat	7	21:43 ± 00:33	06:15 ± 00:27	8.5 ± 0.3	7.3 ± 0.7
3	Flat	8	22:06 ± 00:26	07:06 ± 00:26	9.0 ± 0.7	8.0 ± 0.7
-	Transfer	7	22:10 ± 00:39	07:14 ± 01:30	9.1 ± 1.5	7.7 ± 1.2
4	Hilly	8	22:21 ± 00:37	06:54 ± 00:16	8.5 ± 0.6	7.5 ± 0.4
5	Hilly	8	22:26 ± 00:52	06:16 ± 00:15	7.8 ± 0.9	6.9 ± 0.6
6	Hilly	8	22:28 ± 00:57	06:45 ± 00:11	8.3 ± 0.9	7.2 ± 0.7
7	Mountain	8	22:04 ± 00:26	06:25 ± 00:18	8.3 ± 0.5	7.0 ± 1.1
8	Hilly	7	22:10 ± 01:09	06:37 ± 00:50	8.4 ± 1.3	7.3 ± 1.1
9	Mountain	7	22:01 ± 00:33	06:40 ± 00:14	8.7 ± 0.6	7.5 ± 0.2
-	Rest	7	22:01 ± 00:57	06:45 ± 00:21	8.7 ± 0.9	7.2 ± 1.7
10	Hilly	7	22:37 ± 01:36	06:04 ± 00:28	7.4 ± 1.5	6.4 ± 1.3
11	Mountain	7	21:56 ± 00:23	06:48 ± 00:21	8.9 ± 0.4	7.7 ± 0.6
12	Mountain	7	22:14 ± 00:47	06:20 ± 00:28	8.1 ± 0.6	7.0 ± 0.7
13	Flat	6	21:54 ± 00:41	06:47 ± 00:17	8.8 ± 0.8	7.8 ± 0.8
14	Hilly	6	22:16 ± 00:34	06:43 ± 00:12	8.4 ± 0.6	7.3 ± 0.8
15	Flat	6	22:14 ± 00:47	06:11 ± 00:16	7.9 ± 0.9	6.8 ± 0.7
-	Rest	6	21:51 ± 00:23	06:26 ± 00:29	8.6 ± 0.3	7.5 ± 0.5
16	Hilly	5	22:02 ± 00:41	06:49 ± 00:30	8.8 ± 0.9	7.5 ± 0.7
17	Mountain	6	22:28 ± 00:31	06:51 ± 00:35	8.4 ± 0.7	7.1 ± 0.3
18	Mountain	6	22:04 ± 00:46	06:34 ± 00:22	8.5 ± 0.7	7.0 ± 0.5
19	Flat	5	22:13 ± 00:40	06:25 ± 00:19	8.2 ± 0.8	7.0 ± 0.8
20	Time Trial	6	22:55 ± 00:58	06:44 ± 00:25	7.8 ± 0.8	6.5 ± 0.8
21	Flat	3	01:14 ± 00:47	06:48 ± 01:70	5.6 ± 2.0	5.1 ± 2.0
Post-race	-	6	22:18 ± 00:26	06:03 ± 00:29	7.7 ± 0.5	6.9 ± 0.6

Data are mean ± standard deviation

TIB = time in bed, TST = total sleep time

Table 3A Sleep efficiency, light sleep, slow wave sleep, and rapid eye movement sleep for baseline, each stage of the race, and post-race for male cyclists competing in the 2022 Tour de France

Day/Stage	Classification	Cyclists (n)	SE (%)	Light Sleep (%)	SWS Sleep (%)	REM Sleep (%)
Baseline	-	8	87.0 ± 6.4	45.1 ± 7.9	18.5 ± 3.6	23.4 ± 6.1
1	Time Trial	8	88.4 ± 8.8	45.3 ± 5.6	17.4 ± 4.0	25.7 ± 7.5
2	Flat	7	85.4 ± 7.0	46.3 ± 9.9	15.8 ± 5.0	23.4 ± 5.1
3	Flat	8	88.8 ± 4.9	47.2 ± 12.4	19.6 ± 2.7	22.0 ± 8.8
-	Transfer	7	85.2 ± 8.8	48.4 ± 12.5	18.1 ± 3.9	18.8 ± 9.8
4	Hilly	8	87.9 ± 4.8	50.0 ± 11.9	18.1 ± 4.5	19.8 ± 7.1
5	Hilly	8	88.3 ± 5.2	51.3 ± 9.5	17.6 ± 4.5	19.4 ± 6.0
6	Hilly	8	87.3 ± 5.2	49.8 ± 12.1	17.8 ± 4.1	19.8 ± 9.0
7	Mountain	8	83.9 ± 13.5	45.1 ± 12.3	17.4 ± 5.3	21.3 ± 10.7
8	Hilly	7	86.7 ± 9.5	46.6 ± 12.5	17.4 ± 2.5	22.7 ± 7.8
9	Mountain	7	87.2 ± 3.9	51.7 ± 14.2	14.8 ± 5.6	20.7 ± 9.1
-	Rest	7	82.7 ± 17.8	49.0 ± 16.9	17.7 ± 4.4	16.0 ± 11.2
10	Hilly	7	86.2 ± 2.6	52.5 ± 15.3	16.4 ± 6.1	17.2 ± 11.4
11	Mountain	7	87.3 ± 6.4	49.8 ± 8.4	17.1 ± 2.0	20.3 ± 9.5
12	Mountain	7	85.9 ± 4.2	51.1 ± 6.3	17.4 ± 5.7	17.4 ± 5.5
13	Flat	6	88.6 ± 4.1	53.1 ± 12.0	17.1 ± 5.0	18.4 ± 8.9
14	Hilly	6	86.6 ± 8.1	51.9 ± 14.3	18.6 ± 6.3	16.1 ± 11.0
15	Flat	6	86.1 ± 7.7	51.5 ± 11.0	17.5 ± 3.9	17.1 ± 9.3
-	Rest	6	87.6 ± 4.8	53.9 ± 10.7	16.1 ± 5.0	17.6 ± 8.6
16	Hilly	5	86.4 ± 8.9	52.3 ± 12.3	15.5 ± 2.8	18.6 ± 12.7
17	Mountain	6	85.2 ± 6.8	44.6 ± 10.9	19.1 ± 2.7	21.5 ± 12.1
18	Mountain	6	83.3 ± 8.7	49.8 ± 5.9	17.5 ± 7.3	16.0 ± 9.6
19	Flat	5	85.6 ± 7.1	52.4 ± 10.2	14.2 ± 3.7	19.1 ± 8.3
20	Time Trial	6	84.0 ± 8.6	48.3 ± 15.4	17.1 ± 3.7	18.6 ± 9.2
21	Flat	3	90.2 ± 5.0	52.2 ± 12.0	19.5 ± 7.1	18.4 ± 8.5
Post-race	-	6	89.6 ± 5.0	49.7 ± 6.2	18.9 ± 3.2	21.1 ± 3.6

Data are mean ± standard deviation

SE = sleep efficiency, SWS = slow wave sleep, REM = rapid eye movement

Table 4A Resting heart rate and heart rate variability for baseline, each stage of the race, and post-race for male cyclists competing in the 2022 Tour de France

Day/Stage	Classification	Cyclists (n)	RHR (beats·min^{-1})	HRV-rMSSD (ms)
Baseline	-	8	41.8 ± 6.5	108.5 ± 24.6
1	Time Trial	8	40.9 ± 5.7	106.4 ± 23.6
2	Flat	7	44.6 ± 7.9	97.7 ± 25.9
3	Flat	8	43.6 ± 7.2	98.8 ± 17.4
-	Transfer	7	40.9 ± 6.6	114.9 ± 27.8
4	Hilly	8	42.5 ± 6.5	105.6 ± 21.3
5	Hilly	8	43.4 ± 6.9	100.3 ± 25.7
6	Hilly	8	46.1 ± 8.4	92.5 ± 25.6
7	Mountain	8	45.5 ± 8.2	99.5 ± 28.0
8	Hilly	7	44.7 ± 8.4	93.4 ± 25.2
9	Mountain	7	46.7 ± 9.1	92.8 ± 33.5
-	Rest	7	42.1 ± 6.9	107.3 ± 39.3
10	Hilly	7	45.9 ± 7.7	96.6 ± 24.0
11	Mountain	7	45.7 ± 8.8	89.6 ± 26.4
12	Mountain	7	48.0 ± 9.3	81.9 ± 29.1
13	Flat	6	45.3 ± 8.5	99.6 ± 29.5
14	Hilly	6	47.3 ± 8.9	90.4 ± 28.3
15	Flat	6	46.0 ± 8.2	98.2 ± 30.8
-	Rest	6	41.8 ± 6.7	119.3 ± 26.8
16	Hilly	5	44.0 ± 8.7	113.8 ± 13.9
17	Mountain	6	45.8 ± 7.4	96.2 ± 28.9
18	Mountain	6	49.3 ± 7.8	75.1 ± 22.6
19	Flat	5	41.4 ± 6.1	102.7 ± 31.7
20	Time Trial	6	42.0 ± 7.9	116.0 ± 29.4
21	Flat	3	47.7 ± 13.7	87.5 ± 40.6
Post-race	-	6	44.0 ± 8.6	107.1 ± 20.0

Data are mean ± standard deviation

RHR = resting heart rate, HRV = heart rate variability, rMSSD = root mean square of successive differences

Table 5A TRIMP, daily strain, sleep and cardiac variables for each week of the race in male cyclists competing in the 2022 Tour de France

Variable	Baseline	Week 1	Week 2	Week 3
TRIMP (au)	-	570.9 ± 150.0 (n=55)	581.7 ± 143.2 (n=39)	522.4 ± 133.5 (n=27)
Daily strain (au)	13.1 ± 3.7 (n=8)	19.5 ± 2.1 (n=59)	19.8 ± 1.1 (n=39)	19.7 ± 1.3 (n=22)
Sleep onset (hh:mm)	22:03 ± 00:59 (n=8)	22:11 ± 00:43 (n=69)	22:12 ± 00:53 (n=39)	22:22 ± 00:46 (n=28)
Sleep offset (hh:mm)	06:21 ± 01:02 (n=8)	06:35 ± 00:30 (n=69)	06:28 ± 00:28 (n=39)	06:41 ± 00:27 (n=28)
Time in bed (h)	8.3 ± 0.3 (n=8)	8.4 ± 0.8 (n=69)	8.3 ± 1.0 (n=39)	8.3 ± 0.8 (n=28)
Total sleep time (h)	7.2 ± 0.4 (n=8)	7.3 ± 0.8 (n=69)	7.2 ± 1.0 (n=39)	7.0 ± 6.7 (n=28)
Sleep efficiency (%)	87.0 ± 6.4 (n=8)	87.1 ± 7.3 (n=69)	86.8 ± 5.5 (n=39)	84.8 ± 7.6 (n=28)
Light sleep (%)	45.1 ± 7.9 (n=8)	48.1 ± 11.0 (n=69)	51.6 ± 10.8 (n=39)	49.3 ± 10.9 (n=28)
Slow wave sleep (%)	18.5 ± 3.6 (n=8)	17.4 ± 4.3 (n=69)	17.3 ± 4.7 (n=39)	16.8 ± 4.4 (n=28)
REM sleep (%)	23.4 ± 6.1 (n=8)	21.6 ± 7.9 (n=69)	17.8 ± 8.9 (n=39)	18.7 ± 9.9 (n=28)
Resting HR (beats·min^{-1})	41.8 ± 6.5 (n=8)	44.2 ± 7.4 (n=69)	46.4 ± 8.1 (n=39)	44.6 ± 7.7 (n=28)
HRV-rMSSD (ms)	108.5 ± 24.6 (n=8)	98.7 ± 24.3 (n=69)	92.5 ± 26.8 (n=39)	100.2 ± 28.7 (n=28)

Data are mean ± standard deviation

n = the number of observations

TRIMP = training impulse, au = arbitrary units, REM = rapid eye movement, HR = heart rate, HRV = heart rate variability, rMSSD = root mean square of successive differences

Table 6A TRIMP, daily strain, sleep and cardiac variables across stage classifications in male cyclists competing in the 2022 Tour de France

Variable	Rest	Flat	Hilly	Mountain	Time Trial
TRIMP (au)	-	451.9 ± 143.1 (n=33)	592.9 ± 132.2 (n=46)	625.8 ± 108.0 (n=39)	-
Daily strain (au)	13.1 ± 4.1 (n=21)	18.5 ± 1.7 (n=35)	19.8 ± 2.0 (n=48)	20.3 ± 0.6 (n=40)	-
Sleep onset (hh:mm)	22:01 ± 00:41 (n=20)	22:02 ± 00:37 (n=32)	22:21 ± 00:56 (n=49)	22:07 ± 00:34 (n=41)	22:31 ± 00:52 (n=14)
Sleep offset (hh:mm)	06:49 ± 00:58 (n=20)	06:34 ± 00:31 (n=32)	06:35 ± 00:30 (n=49)	06:36 ± 00:25 (n=41)	06:30 ± 00:34 (n=14)
Time in bed (h)	8.8 ± 1.0 (n=20)	8.5 ± 0.8 (n=32)	8.2 ± 1.0 (n=49)	8.5 ± 0.6 (n=41)	8.0 ± 0.9 (n=14)
Total sleep time (h)	7.5 ± 1.2 (n=20)	7.4 ± 0.8 (n=32)	7.1 ± 0.9 (n=49)	7.2 ± 0.7 (n=41)	6.9 ± 0.9 (n=14)
Sleep efficiency (%)	85.1 ± 11.6 (n=20)	87.0 ± 6.0 (n=32)	87.1 ± 6.1 (n=49)	85.5 ± 7.8 (n=41)	86.5 ± 8.7 (n=14)
Light sleep (%)	50.3 ± 13.3 (n=20)	49.7 ± 10.9 (n=32)	50.5 ± 11.9 (n=49)	48.7 ± 10.0 (n=41)	46.6 ± 10.5 (n=14)
Slow wave sleep (%)	17.4 ± 4.3 (n=20)	17.0 ± 4.2 (n=32)	17.4 ± 4.4 (n=49)	17.2 ± 4.9 (n=41)	17.3 ± 3.7 (n=14)
REM sleep (%)	17.4 ± 9.5 (n=20)	20.3 ± 8.0 (n=32)	19.2 ± 8.8 (n=49)	19.6 ± 9.2 (n=41)	22.7 ± 8.8 (n=14)
Resting HR (beats·min^{-1})	41.6 ± 6.4 (n=20)	44.3± 7.3 (n=32)	44.8 ± 7.6 (n=49)	46.8 ± 8.1 (n=41)	41.4 ± 6.5 (n=14)
HRV-rMSSD (ms)	113.6 ± 30.8 (n=20)	99.2 ± 25.0 (n=32)	98.5 ± 23.7 (n=49)	89.6 ± 27.8 (n=41)	110.5 ± 25.6 (n=14)

Data are mean ± standard deviation

n = the number of observations

TRIMP = training impulse, au = arbitrary units, REM = rapid eye movement, HR = heart rate, HRV = heart rate variability, rMSSD = root mean square of successive differences

Table 7A TRIMP and Daily Strain for baseline, each stage of the race, and post-race for female cyclists competing in the 2022 Tour de France Femme

Day/Stage	Classification	TRIMP (au)	Daily Strain (au)
Baseline	-	-	15.3 ± 1.9 (n=9)
1	Flat	478.0 ± 43.8 (n=9)	20.4 ± 0.3 (n=9)
2	Flat	605.8 ± 57.3 (n=9)	20.5 ± 0.2 (n=9)
3	Hilly	622.1 ± 39.7 (n=9)	20.5 ± 0.2 (n=9)
4	Hilly	596.5 ± 55.5 (n=9)	20.5 ± 0.2 (n=9)
5	Flat	644.5 ± 68.2 (n=8)	20.3 ± 0.3 (n=9)
6	Hilly	550.1 ± 29.6 (n=8)	20.4 ± 0.2 (n=8)
7	Mountain	794.2 ± 53.3 (n=7)	20.6 ± 0.1 (n=7)
8	Mountain	638.6 ± 54.0 (n=7)	20.5 ± 0.2 (n=7)
Post-race	-	-	13.5 ± 2.2 (n=9)

Data are mean ± standard deviation

n = the number of participants

TRIMP = training impulse, au = arbitrary units

Table 8A Sleep onset, sleep offset, time in bed and total sleep time for baseline, each stage of the race, and post-race for female cyclists competing in the 2022 Tour de France Femme

Day/Stage	Classification	Cyclists (n)	Sleep Onset (hh:mm)	Sleep Offset (hh:mm)	TIB (h)	TST (h)
Baseline	-	9	21:18 ± 00:42	05:58 ± 00:30	8.7 ± 0.6	7.7 ± 0.5
1	Flat	9	20:48 ± 00:37	05:59 ± 00:32	9.2 ± 0.7	8.1 ± 0.5
2	Flat	9	21:25 ± 00:39	05:58 ± 00:28	8.6 ± 1.0	7.5 ± 0.8
3	Hilly	9	21:13 ± 00:49	06:14 ± 00:29	9.0 ± 0.9	7.9 ± 0.5
4	Hilly	9	21:13 ± 00:48	05:29 ± 00:31	8.3 ± 0.6	7.4 ± 0.5
5	Flat	8	21:29 ± 00:28	05:52 ± 00:40	8.4 ± 0.7	7.7 ± 0.6
6	Hilly	8	21:15 ± 00:40	06:20 ± 00:41	9.1 ± 1.0	8.1 ± 0.6
7	Mountain	8	21:46 ± 00:42	06:06 ± 00:34	8.3 ± 1.0	7.6 ± 0.9
8	Mountain	7	00:22 ± 01:24	06:33 ± 01:35	6.2 ± 2.3	5.5 ± 1.9
Post-race	-	9	21:14 ± 00:40	06:17 ± 00:32	8.5 ± 0.5	7.7 ± 0.5

Data are mean ± standard deviation

TIB = time in bed, TST = total sleep time

Table 9A Sleep efficiency, light sleep, slow wave sleep, and rapid eye movement sleep for baseline, each stage of the race, and post-race for female cyclists competing in the 2022 Tour de France Femme

Day/Stage	Classification	Cyclists (n)	SE (%)	Light Sleep (%)	SWS Sleep (%)	REM Sleep (%)
Baseline	-	9	88.8 ± 4.0	41.9 ± 5.5	20.2 ± 2.7	26.7 ± 6.1
1	Flat	9	88.8 ± 4.7	43.4 ± 5.2	19.4 ± 5.1	26.0 ± 7.0
2	Flat	9	88.1 ± 3.1	43.6 ± 8.6	20.1 ± 4.2	24.4 ± 7.8
3	Hilly	9	87.9 ± 7.1	40.5 ± 7.6	19.1 ± 6.2	28.2 ± 8.6
4	Hilly	9	89.7 ± 5.3	51.8 ± 11.7	18.9 ± 6.6	19.1 ± 8.3
5	Flat	8	91.7 ± 3.6	44.8 ± 11.6	20.9 ± 3.6	26.0 ± 10.4
6	Hilly	8	89.3 ± 6.8	43.1 ± 9.6	19.9± 6.0	26.3 ± 9.9
7	Mountain	8	91.1 ± 45	55.0 ± 13.3	18.0 ± 6.9	18.1 ± 8.3
8	Mountain	7	90.5 ± 2.9	56.0 ± 10.8	17.5 ± 7.1	17.0 ± 7.7
Post-race	-	9	90.5 ± 4.2	44.9 ± 5.1	20.2 ± 2.0	25.4 ± 4.8

Data are mean ± standard deviation

SE = sleep efficiency, SWS = slow wave sleep, REM = rapid eye movement

Table 10A Resting heart rate and heart rate variability for baseline, each stage of the race, and post-race for female cyclists competing in the 2022 Tour de France Femme

Day/Stage	Classification	Cyclists (n)	RHR (beats·min^{-1})	HRV-rMSSD (ms)
Baseline	-	9	45.8 ± 7.6	119.8 ± 40.4
1	Flat	9	46.2 ± 6.5	113.4 ± 34.5
2	Flat	9	48.2 ± 7.5	115.7 ± 39.7
3	Hilly	9	51.4 ± 8.1	106.1 ± 45.7
4	Hilly	9	50.7 ± 8.9	112.8 ± 53.3
5	Flat	8	51.0 ± 8.5	115.1 ± 56.9
6	Hilly	8	50.1 ± 8.8	120.4 ± 57.3
7	Mountain	8	51.8 ± 8.0	120.7 ± 50.9
8	Mountain	7	52.9 ± 10.9	110.5 ± 56.0
Post-race	-	9	45.0 ± 6.6	113.6 ± 33.6

Data are mean ± standard deviation

RHR = resting heart rate, HRV = heart rate variability, rMSSD = root mean square of successive differences

Table 11A TRIMP, daily strain, sleep and cardiac variables across stage classifications in female cyclists competing in the 2022 Tour de France Femme

Variable	Flat	Hilly	Mountain
TRIMP (au)	573.5 ± 90.8 (n=26)	591.1 ± 51.3 (n=26)	716.4 ± 95.6 (n=14)
Daily strain (au)	20.4 ± 0.3 (n=27)	20.5 ± 0.2 (n=26)	20.6 ± 0.2 (n=14)
Sleep onset (hh:mm)	21:13 ± 00:38 (n=26)	21:14 ± 00:44 (n=26)	21:46 ± 00:42 (n=8)
Sleep offset (hh:mm)	05:57 ± 00:32 (n=26)	06:01 ± 00:40 (n=26)	06:05 ± 00:34 (n=8)
Time in bed (h)	8.7 ± 0.9 (n=26)	8.8 ± 0.9 (n=26)	8.3 ± 1.0 (n=8)
Total sleep time (h)	7.8 ± 0.7 (n=26)	7.8 ± 0.6 (n=26)	7.6 ± 0.9 (n=8)
Sleep efficiency (%)	89.4 ± 4.0 (n=26)	89.0 ± 6.2 (n=26)	91.1 ± 4.5 (n=8)
Light sleep (%)	43.9 ± 8.4 (n=26)	45.2 ± 10.6 (n=26)	55.0 ± 13.3 (n=8)
Slow wave sleep (%)	20.1 ± 4.2 (n=26)	19.3 ± 6.0 (n=26)	18.0 ± 6.9 (n=8)
REM sleep (%)	25.5 ± 8.2 (n=26)	24.5 ± 9.5 (n=26)	18.1 ± 8.4 (n=8)
Resting HR (beats·min^{-1})	48.4 ± 7.5 (n=26)	50.8 ± 8.3 (n=26)	51.8 ± 8.0 (n=8)
HRV-rMSSD (ms)	114.7 ± 42.3 (n=26)	112.8 ± 50.3 (n=26)	120.7 ± 50.9 (n=8)

Data are mean ± standard deviation

n = the number of observations

TRIMP = training impulse, au = arbitrary units, REM = rapid eye movement, HR = heart rate, HRV = heart rate variability, rMSSD = root mean square of successive differences
